# Do People Feel Less Lonely When Alone if They Use Media? Findings From Two Experience Sampling Studies

**DOI:** 10.1093/geroni/igaf122.368

**Published:** 2025-12-31

**Authors:** Gizem Hueluer, Christina Ristl, Jana Nikitin

**Affiliations:** University of Bamberg, Bamberg, Germany; University of Vienna, Vienna, Wien, Austria; University of Vienna, Vienna, Wien, Austria

## Abstract

Solitude is a common daily experience that can have both negative and positive impacts on well-being depending on individual and situational factors. In this study, we examined whether media use moderates the relationship between being alone and loneliness, with a particular focus on whether this effect varies by age. To do so, we used data from two experience sampling studies, which were analyzed using multilevel regression models. In both studies, participants reported higher loneliness when alone. Media use moderated this effect in both studies: In the first study (N = 103, 55% women, mean age = 47 years, SD = 18 years, range = 19 to 79 years), media use was associated with higher overall loneliness, but it was associated with lower loneliness while being alone, particularly in older adults. The findings of the second study (N = 265, 56% women, mean age = 48 years, SD = 18 years, range = 17 to 83 years) were more nuanced: While using a computer or the internet was associated with lower loneliness when alone, watching TV was linked to greater loneliness when alone. These effects were not moderated by age. Reading and listening to the radio had no significant effects. These findings suggest that while some forms of media use may buffer against loneliness when alone, others may exacerbate it, and the effects may vary based on media type and age. The results highlight the importance of considering the specific ways in which individuals engage with media when addressing loneliness.

